# Contributing factors to acute malnutrition among children admitted to public health facilities in South West Ethiopia: a matched case–control study

**DOI:** 10.3389/fnut.2025.1525838

**Published:** 2025-02-24

**Authors:** Dessalegn Tamiru, Shimelis Girma, Getu Gizaw

**Affiliations:** Department of Nutrition and Dietetics, Jimma University, Jimma, Ethiopia

**Keywords:** malnutrition, child feeding, public health, Jimma town, undernutrition

## Abstract

**Introduction:**

In Ethiopia, acute malnutrition is one of the potential challenges to achieving the United Nations’ Sustainable Development Goals in reducing child mortality. Thus, this study aimed to determine factors associated with acute malnutrition among children aged 6–59 months attending public health facilities in Jimma town, South West Ethiopia, from March to December 2017.

**Methods:**

An institution-based age-matched case–control study design was used. Two hundred and thirty-four children aged 6 to 59 months were randomly selected. Data were analyzed using SPSS version 20. Variables with a p-value of ≤0.25 in the bivariate analyses were entered into a multivariable regression analysis to determine the independent predictors of acute malnutrition.

**Results:**

This study showed that lack of maternal education (AOR = 4.08, 95% CI, 1.46, 11.40), poor child feeding (AOR = 5.97, 95% CI, 1.83, 19.44), low wealth index (AOR = 3.76, 95% CI, 1.24, 11.38), less hand washing (AOR = 5.57, 95% CI, 1.82, 16.97), exposure to diarrhea (AOR = 3.58, 95% CI, 1.15, 11.07), and bottle-feeding (AOR = 3.98, 95% CI, 1.29, 12.36) were significantly associated with acute malnutrition among children attending public health facilities in Jimma town.

**Conclusion:**

The findings of this study indicated that the sex of the child, family size, household wealth index, bottle-feeding, and maternal knowledge of child feeding were found to be independent predictors of acute malnutrition. Therefore, emphasis should be given to strengthening caregivers’ socioeconomic status and improving the knowledge of mothers regarding childfeeding practices.

## Background

Adequate provision of nutrients from the early stages of life is crucial to ensure good physical and mental development and long-term health. The double burden of malnutrition is an increasing public health problem worldwide (1). Acute malnutrition is a devastating disease of epidemic proportions. Globally, acute malnutrition continues to threaten the lives of an estimated 7.7% or approximately 52 million children younger than 5; more than two-thirds (69%) live in Asia, and more than one-quarter (29%) live in Africa. In Africa, 14 million children under 5 years are wasted, of whom 4.1 million are severely wasted and 4.2 million are from Eastern African countries (2). Ethiopia has made encouraging progress in reducing malnutrition over the past decades. However, the baseline levels of malnutrition remain so high that the country must continue to make significant investments in nutrition. Accordingly, the proportion of children underweight decreased more substantially by 39% over the period between 2000 and 2014 (3). However, there was only a small decline in the prevalence of wasting over the last 15 years. Recent data indicate that 10% of Ethiopian children are wasted, and 3% are severely wasted (3, 4).

Malnutrition is categorized into severe and moderate acute malnutrition according to the degree of wasting and the presence of edema. Severe acute malnutrition is defined as severe wasting (WFH <70% of the median of the WHO child growth standards or WFH < -3 *z*-score using the WHO child growth standards) (5–7). Acute malnutrition is defined as moderate acute malnutrition if weight-for-height is between 70 and 80% of the median of the WHO child growth standards or weight-for-height < minus 2 z-score using the WHO child growth standards, and edematous cases are always classified as severe (5, 6). Another criterion used to define and classify acute malnutrition is mid-upper arm circumference (MUAC). By using MUAC, acute malnutrition is defined as a child with MUAC < 110 mm, and moderate acute malnutrition is defined as MUAC ≥ 110 mm and < 125 mm (8). MUAC cutoff points are controversial, and the World Health Organization recommends that countries have their own cutoff points to define acute malnutrition; accordingly, Ethiopia sets MUAC cutoff points below 110 mm to define SAM and 110 mm to less than 120 mm to define MAM (9).

In Africa, hunger is estimated to result in a cost of $ 154 million in 2009 for 4.4 million additional clinical episodes associated with undernutrition in children aged 5 years and younger. The study estimated that Ethiopia has lost approximately $ 4.7 billion as the result of undernutrition which is equivalent to 16.5% of its gross domestic product (GDP) (10).

Acute malnutrition is a major public health issue, with its prevalence varying across different regions due to varying local determinants. Ethiopia has one of the highest rates of child undernutrition in sub-Saharan Africa. However, an understanding of the underlying causes remains insufficient. It is crucial to better document the key factors contributing to child malnutrition in Ethiopia and to assess their relative significance. In addition, identifying these determinants can significantly enhance the effectiveness and efficiency of interventions. Therefore, this study aims to identify the factors contributing to acute malnutrition among children admitted to public hospitals in Jimma town, South West Ethiopia.

## Materials and methods

### Study setting and sampling

An institution-based case–control study was carried out among children aged 6–59 months attending public health facilities in Jimma town from March to December 2017. Jimma town is located 350 km from Addis Ababa in the south-west direction and covers a total area of 4,623 hectares. The town has a latitude and longitude of 7^0^ 40’N 36^0^50′E. The daily mean temperature of the town ranges from 20°C to 25°C year-round, and the average annual rainfall is 1,500 mm. In the Jimma zone, cereals contribute to 88.9% of the grain crop area and 93.1% of the production. Pulses cover 8.4% of the grain crop area. There are four public health centers, one zonal hospital, and one specialized medical center, delivering healthcare services for acutely malnourished children. All public health facilities were included in the study, and cases were allocated proportionally based on the average monthly child follow-up.

### Inclusion and exclusion criteria

Cases were all randomly selected children aged 6 to 59 months who had acute malnutrition (weight-for-height z-score below -2SD or below 80% of the median of the WHO child growth standards or by mid-upper arm circumference (MUAC) < 125 mm), while controls were all randomly selected children aged 6 to 59 months and those without acute malnutrition (weight-for-height z-score above -2SD or ≥ 80% of the median of the WHO child growth standards or by mid-upper arm circumference (MUAC) ≥ 125 mm). Age was also considered an inclusion criterion, with the following age intervals used for matching: 6–11 months, 12–23 months, 24–35 months, 36–47 months, and 48–59 months.

### Sample size determination

The sample size was calculated using the STATCALC application in Epi Info 7 with the following assumptions: 46.9% of control households and 68.14% of case households had a family size of five or more, a 95% confidence interval, 80% power, a 2:1 control-to-case ratio, and an odds ratio of 2.42. A 10% non-response rate was added, and the final sample size was 234 (78 cases and 156 controls).

### Data collection and measurements

Semistructured interviewer-administered questionnaires and anthropometric measurement tools (MUAC tape, stadiometer, digital weight scale, and Salter scale) were used to collect data. Questionnaires were prepared in English and translated into Amharic and Afaan Oromo by language experts. The food security status of households was determined based on nine standard household food insecurity (HFIAS) questions that were validated for use in developing countries (11). Data collected on food consumption were used to calculate the dietary diversity score (DDS) and the minimum meal frequency (MMF), which were based on qualitative data from the last 24-h dietary recall preceding the survey. Anthropometric measurements were taken twice with different measures for each child. Weight was measured using a digital electronic measuring scale (SECA) to the nearest 0.1 kg after calibration, barefoot and with as little clothing as possible. Height/length was measured using the standard procedure (Frankfurt position) in a standing position using a height measurement board to the nearest 0.1 cm for children 24 months of age and older. For children under the age of 24 months, height/length was measured in a lying position. Mid-upper arm circumference (MUAC) was measured with an armband/tape using an easily portable measurement device on the left-hand side, halfway between the olecranon and acromion.

### Data processing and analysis

Data were coded and entered into EpiData version 3.1 and exported to SPSS version 20.0 statistical software for analysis. Descriptive statistics were presented using standard statistical parameters such as percentages, means, and standard deviations. Bivariate analysis was conducted, and all explanatory variables that had an association with the outcome variables with a *p*-value less than 0.25 were included in the multivariable analysis. Multi-variable conditional logistic regression analysis was used to determine the independent determinant factor. The wealth index was determined based on the ownership of fixed assets, and the factor score was generated through principal component analysis after checking for assumptions. The score on the household food insecurity access scale (0–27) for each household was summed to produce an index of household food insecurity. Model fitness was assessed using the Hosmer–Lemeshow test, and it was found to be 0.75. Multicollinearity was checked using the variance inflation factor (VIF) and the tolerance test. The result of the VIF was found to be <2, and the tolerance test was greater than 0.1.

## Results

A total of 234 participants were included in the study, consisting of 78 cases and 156 controls. The mean age of the participants was 18.29 ± 10.23 months for the cases and 16.6 ± 10.2 months for the controls. Regarding weight, the average for the cases was 6.6 ± 1.7 kg, while for the controls it was 9.0 ± 2.9 kg (Table 1).

**Table 1:** Sociodemographic characteristics of study population attending public health facilities of Jimma town, South West Ethiopia, March 2017.

Study variables	Case (*n* = 78) *N* (%)	Control (*n* = 156) *N* (%)	*P*-value
**Child sex**			< 0.001
Female	53 (67.9)	57 (36.5)	
Male	25 (32.1)	99 (63.5)	
**Place of residence**			< 0.001
Rural	56 (71.8)	47 (30.1)	
Urban	22 (28.2)	109 (69.9)	
**Birth order**			< 0.001
> 3	53 (67.9)	43 (27.6)	
1–3	25(32.1)	113 (72.4)	
**Maternal age**			< 0.001
15–24	6 (5.0)	9 (7.4)	
25–34	52 (43.0)	43 (35.5)	
35–44	31 (25.6)	37 (30.6)	
**Maternal education**			< 0.001
No formal education	54 (69.2)	48 (30.8)	
Formal education	24 (30.8)	108 (69.2)	
**Parental marital status**			< 0.002
Divorced/Widowed	13 (16.7)	7 (4.5)	
Married	65 (83.3)	149 (95.5)	
**Family size**			< 0.001
Above five	42 (53.8)	21 (13.5)	
Five and less	36 (46.2)	135 (86.5)	
**Wealth index**			< 0.001
Lowest	45 (57.7)	29 (18.6)	
Middle	5 (6.4)	26 (16.7)	
Highest	28 (35.9)	101 (64.7)	
**HFIAS status**			< 0.001
Food insecure	57 (73.1)	57 (36.5)	
Food secure	21 (26.9)	99 (63.5)	
**Solid waste disposal site**			< 0.001
Other than waste pit	39 (50.0)	37 (23.7)	
Solid waste pit	39 (50.0)	119 (76.3)	
**Liquid waste disposal site**			< 0.001
Other than waste pit	48 (61.5)	39 (25.0)	
Liquid waste pit	30 (38.5)	117 (75.0)	
**Mother hand washing practice**			< 0.001
Less frequently	48 (61.5)	32 (20.5)	
More frequently	30 (38.5)	124 (79.5)	

This study showed that 34 (43.6%) of the cases and 20 of the controls (12.8%) had a history of diarrhea in the last 2 weeks prior to data collection. Similarly, approximately two-thirds of the study participants (both cases and controls) had been exclusively breastfed for 6 months (Table 2).

**Table 2:** Maternal and child health care related factors among study population attending public health facilities of Jimma town, South-West Ethiopia, March 2017.

Study variables	Case (*n* = 78) *N* (%)	Control (*n* = 156) *N* (%)	*P*-value
**Place of delivery**			0.249
Home Health institution	15 (19.2) 63 (80.8)	21 (13.5) 135 (86.5)	
**ANC**			0.628
No Yes	8 (10.3) 70 (89.7)	13 (8.3) 143 (91.7)	
**History of diarrhoea**			< 0.001
Yes No	34 (43.6) 44 (56.4)	20 (12.8) 136 (87.2)	
**History of febrile illness**			0.284
Yes No	23 (29.5) 55 (70.5)	57 (36.5) 99 (63.5)	
**Vaccination status**			0.148
Incomplete Completed	18 (23.1) 60 (76.9)	24 (15.4) 132 (84.6)	
**Breast feeding initiation**			0.269
> 1 h ≤ 1 h	21 (26.9) 57 (73.1)	32 (20.5) 124 (79.5)	
**Perlacteal feed**			0.114
Yes No	18 (23.1) 60 (76.9)	23 (14.7) 133 (85.3)	
**Colostrum**			0.571
Discarded Given to child	4 (5.1) 74 (94.9)	11 (7.1) 145 (92.9)	
**Duration of EBF**			0.900
< or > than 6 months For 6 months	22 (28.2) 56 (71.8)	42 (26.9) 114 (73.1)	
**DDS**			0.001
Low DDS High DDS	71 (91.0) 7 (9.0)	111 (71.2) 45 (28.8)	
**Bottle feeding**			< 0.001
Yes No	37 (47.4) 41 (52.6)	35 (22.4) 121 (77.6)	
**Way of feeding**			0.714
All children together With separate dish	6 (7.7) 72 (92.3)	10 (6.4) 146 (93.6)	
**Maternal knowledge**			<0.001
Poor Fair Good	45 (57.7) 18 (23.1) 15 (19.2)	48 (30.8) 21 (13.5) 87 (55.8)	

The results of the multivariable logistic regression analysis identified several factors that were significantly associated with acute malnutrition. These included being a female child (AOR = 2.99, 95% CI, 1.07 to 8.38), belonging to a low wealth index (AOR = 3.76, 95% CI, 1.24 to 11.38), and having a mother with no formal education (AOR = 4.08, 95% CI, 1.46 to 11.40).

This study also showed that having more than five family members (AOR = 3.24, 95% CI, 1.14 to 9.21), infrequent hand washing practices (AOR = 5.57, 95% CI, 1.82 to 16.97), a history of diarrhea (AOR = 3.58, 95% CI, 1.15 to 11.07), bottle-feeding (AOR = 3.98, 95% CI, 1.29 to 12.36), and poor maternal knowledge about child feeding (AOR = 5.97, 95% CI, 1.83 to 19.44) were all found to increase the likelihood of acute malnutrition (Table 3).

**Table 3:** Predictors of acute malnutrition among participants attending public health facilities of Jimma town, South West Ethiopia, 2017.

Study variables	Case (*n* = 78) *N* (%)	Control (*n* = 156) *N* (%)	COR (95% CI)	AOR (95% CI)
**Child sex**				
Female Male	53 (67.9) 25 (32.1)	57 (36.5) 99 (63.5)	33.68 (2.07, 6.55)*** 1 1	2.99 (1.07, 8.38)* 1
**Wealth index**				
Lowest Middle Highest	45 (57.7) 5 (6.4) 28 (35.9)	29 (18.6) 26 (16.7) 101 (64.7)	5.59 (2.99, 10.48)*** 0.69 (0.24, 1.97) 1	3.76 (1.24, 11.38)* 0.49 (0.09, 2.52) 1
**Birth order**				
Above 3 1–3	53 (67.9) 25 (32.1)	43 (27.6) 113 (72.4)	5.57 (3.09, 10.06)*** 1	4.25 (1.44, 12.53)** 1
**Maternal education**				
No formal education Formal education	54 (69.2) 24(30.8)	48 (30.8) 108 (69.2)	5.06 (2.81,9.12)*** 1	4.08 (1.46,11.40)** 1
**Family size**				
Above five Five and less	42 (53.8) 36 (46.2)	21 (13.5) 135 (86.5)	7.5 (3.96, 14.22)*** 1	3.24 (1.14,9.21)* 1
**Liquid waste disposal site**				
Other than waste pit Liquid waste pit	48(61.5) 30(38.5)	39 (25.0) 117 (75.0)	4.8 (2.68, 8.59)*** 1	5.95 (1.83,16.97)** 1
**Mother hand washing**				
Less frequently Frequently	48 (61.5) 30 (38.5)	32 (20.5) 124 (79.5)	6.2 (3.41, 11.29)*** 1	5.57 (1.82,16.97)** 1
**History of diarrhoea**				
Yes No	34 (43.6) 44 (56.4)	20 (12.8) 136 (87.2)	5.25 (2.75,10.05)*** 1	3.58 (1.15,11.07)* 1
**Bottle feeding**				
Yes No	37 (47.4) 41 (52.6)	35 (22.4) 121 (77.6)	3.12 (1.74, 5.59)*** 1	3.98 (1.29,12.36)* 1
**Maternal knowledge**				
Poor Fair Good	45 (57.7) 18 (23.1) 15 (19.2)	48 (30.8) 21 (13.5) 87 (55.8)	5.44 (2.75, 10.76)*** 4.97 (2.16, 11.45)*** 1	5.97 (1.83, 19.44)** 2.94 (0.67,12.80) 1

*p < 0.05; **p < 0.01; ***p < 0.001.

## Discussion

Acute malnutrition is a major cause of death in children in developing countries, and the magnitude of acute malnutrition persists longer without reduction. Although studies with robust designs, like the present one, are the preferred method for identifying the determinants of acute malnutrition and informing strategies for its reduction, there has been limited research on the predictors of acute malnutrition among children aged 6–59 months in the study area. Thus, this study was designed to determine the determinant factors of acute malnutrition among children aged 6–59 months attending public health facilities in Jimma town, South West Ethiopia.

The children whose mothers did not attend formal education were four times more likely to be acutely malnourished than children whose mothers attended formal education which is consistent with the study conducted in North Gondar, West Ethiopia (12–15). However, it was observed that paternal education was not significantly associated with acute malnutrition, which is not consistent with other studies (13, 16). The possible explanation may be the culture and tradition in the study area, where mothers are more responsible for child care than fathers. In addition, fathers of children are usually involved in agriculture and outdoor activities.

This study showed that female children were more likely to be affected by acute malnutrition than their male counterparts, which is consistent with other studies (17–19). The reason is unclear, but it may be because of culture or tradition, which provides a strong buffer for male children which may lead to female children being disadvantaged in terms of care and nutrition.

The findings of this study showed that participants living with more than five family members were more likely exposed to acute malnutrition compared to their counterparts. Studies have also shown that having a large number of family members is a significant contributor to under-five malnutrition (12, 14). This may be because a large family size leads to competition for food or because families with more children experience more economic strain for food consumption and are thus more likely to suffer from poor nutritional status. In other words, inadequate allocation of household resources among many children may lead to low nutritional status.

This study indicated that children from low-wealth index households were more likely to be acutely malnourished than children from households with a high-wealth index. A study conducted in North Shewa indicated that children belonging to the low-income group were at a higher risk of being wasted than children of higher-income families (20, 21). Low income levels in developing nations limit the kinds and amounts of food available for consumption. Low income also increases the likelihood of infection through mechanisms such as inadequate personal and environmental hygiene. On the contrary, households with a high wealth index have greater purchasing capacity for food and other goods needed to ensure the health of children (4, 22, 23).

Children who had diarrhea disease in the last 2 weeks prior to the survey were found to be approximately four times more likely to be exposed to malnutrition compared to children who had no diarrhea. This may be due to excessive loss of fluids and electrolytes, loss of appetite, and failure to absorb food during diarrheal episodes. A similar finding was seen in the studies conducted in Gimbi (16). On the other hand, the present study did not show a significant association between the morbidity status of the child with fever preceding 2 weeks before the onset of acute malnutrition. This is consistent with similar studies in Gimbi and Hidhabu Abote (16, 21).

In terms of feeding and care practices and knowledge, the bottle-fed child was more likely to have acute malnutrition than a non-bottle-fed child. Many studies have shown that bottle-feeding should be discouraged at any age because it is usually associated with an increased risk of illness, especially diarrhea, due to hygienic problems, that is to say, difficulty in sterilizing the teats properly. This study also found a statistically significant difference in knowledge of the recommended optimal child feeding between caregivers of acutely malnourished children and the controls (3, 13).

In this study, household food insecurity was not found to be statistically significantly associated with acute malnutrition. This finding suggests that food security alone may not be sufficient to ensure adequate nutrition. It supports the idea that while households may have access to enough food, this does not necessarily translate into proper nutrition, as food security does not always address the quality or nutritional value of the food consumed. This distinction highlights the need for interventions that focus not just on food availability but also on improving the nutritional content of the diet to prevent malnutrition (5, 9, 17, 23).

The main strength of the study was that the researchers’ use of measures other than data collectors to minimize interviewer bias, and the design used was able to evaluate the association of acute malnutrition with other exposure variables. The findings of this study may have a significant implication for the prevention of acute malnutrition, and emphasis should be given to strengthening caregivers’ socioeconomic status and knowledge regarding child-feeding practices.

However, this study has limitations, such as the reliance on self-reported data for some explanatory variables, which could be subject to recall and social desirability bias. Furthermore, the study did not address environmental enteropathy. In addition, this study was conducted only among hospitalized children and cannot be easily generalized to malnourished children in home or community settings. Hospitalized children often represent more severe cases of malnutrition, and their circumstances, including healthcare access, socioeconomic factors, and food availability, may differ from those in less critical conditions outside of hospitals.

## Conclusion

The findings of this study can be summarized as the sex of the child, family size, birth order, maternal educational status, household wealth index, bottle-feeding, liquid waste disposal, diarrhea, and maternal knowledge were found to be independent predictors of acute malnutrition. In general, emphasis should be given to improving caregivers’ socioeconomic status and their knowledge of childcare practices. In addition, the government should create job opportunities and include low-income households in programs such as safety-net programs to address the issues of undernutrition.

## Data Availability

The raw data supporting the conclusions of this article will be made available by the authors, without undue reservation.
